# Anisotropic Cellular Forces Drive Hexagonal‐to‐Tetragonal Tiling Transitions in the *Drosophila* Eye

**DOI:** 10.1111/dgd.70050

**Published:** 2026-03-10

**Authors:** Ting Zheng, Steven R. Davis, Cuicui Li, Weichao Ren, Makoto Sato

**Affiliations:** ^1^ Graduate School of Frontier Science Initiative Kanazawa University Kanazawa Ishikawa Japan; ^2^ School of Computer and Information Science Inner Mongolia Medical University Hohhot Inner Mongolia China; ^3^ School of Life Science and Technology, Institute of Science Tokyo Tokyo Japan; ^4^ Graduate School of Medical Sciences Kanazawa University Kanazawa Ishikawa Japan; ^5^ Mathematical Neuroscience Unit, Institute for Frontier Science Initiative Kanazawa University Kanazawa Ishikawa Japan

**Keywords:** anisotropic force, cell morphogenesis, *Drosophila* compound eye, vertex model

## Abstract

Tile patterns are fundamental organizational principles of multicellular epithelial tissues. The *Drosophila* compound eye provides a striking example, in which ommatidia are arranged in a highly regular hexagonal lattice, while tetragonal patterns emerge in specific small‐eye mutants. Although increased dorsoventral tension has been implicated in this hexagonal‐to‐tetragonal transition, conventional vertex models fail to reproduce the observed pattern transformation, indicating the presence of additional uncharacterized force‐generating mechanisms. Here, we demonstrate that anisotropic cellular forces driven by radial actin fibers are a key determinant of ommatidial tiling geometry. By extending the vertex model to incorporate both dorsoventral stretching and anisotropic forces that generate rotational torque at cell boundaries, we successfully recapitulate the hexagonal‐to‐tetragonal transition observed in mutant eyes. Experimental disruption of radial actin fibers suppressed tetragonal pattern formation and induced irregular tiling, providing in vivo support for the model predictions. Importantly, in silico analyses further revealed that anisotropic forces play a dual role: while they drive tetragonalization under symmetry‐breaking conditions in mutant eyes, they stabilize regular hexagonal tiling in the wild‐type context. These findings identify anisotropic cellular forces as an essential component of epithelial pattern formation and establish an extended vertex model framework for understanding force‐driven morphogenetic transitions during development.

## 1 Introduction

**FIGURE 1:**
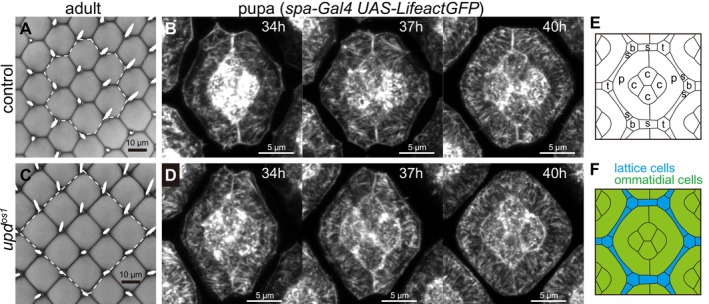
Development of ommatidial patterns. (A, C) Tile patterns of ommatidia in the adult compound eye in control (A) and tetragonal mutant, *upd* ^*os1*^ (C). (B, D) Confocal images of control (B) and *upd* ^*os1*^ mutant eyes (D) at 34, 37, and 40 h APF (*spa‐Gal4 UAS‐LifeactGFP (LaGFP)*), showing the progressive establishment of ommatidial patterns. Actin fibers, visualized via LaGFP, are radially aligned within primary pigment cells. The dark boundaries between ommatidia correspond to ommatidial lattice cells. Scale bars, 10 or 5 μm. (E) Each ommatidium consists of four central cone cells (c), surrounded by two primary pigment cells (p). The surrounding lattice is formed by secondary pigment cells (s), tertiary pigment cells (t), and bristle cells (b), which together contribute to the hexagonal arrangement and maintain optical insulation between adjacent ommatidia. (F) The cone and primary pigment cells are collectively called ommatidial cells (green), while the secondary and tertiary pigment cells and bristle cells are collectively called lattice cells that form the interommatidial boundaries between adjacent ommatidia (blue).

Regular epithelial tiling patterns are a fundamental feature of multicellular tissue organization and are widely observed across animal development. Among these, hexagonal packing is particularly prevalent, as its mechanically stable geometry confers efficient space filling and robustness of the tissue against external perturbations (Honda 1978; Farhadifar et al. 2007; Lecuit and Lenne 2007; Weaire and Phelan 1994). The compound eye of the fruit fly, *Drosophila melanogaster* , serves as a prominent example, comprising approximately 800 ommatidia (Figure 1A), collectively ensuring both structural robustness and optimal optical coverage (Ready et al. 1976; Kumar 2012; Tomlinson 1988; Zelhof et al. 2006; Heberlein and Moses 1995). In the apical epithelial plane, each ommatidial unit comprises four cone cells (c) surrounded by two primary pigment cells (p), collectively called ommatidial cells, forming a hexagonal ommatidium. Surrounding lattice cells, including secondary and tertiary pigment cells and bristle cells (s, t, and b), form the hexagonal interommatidial lattice that delineates the boundaries between adjacent ommatidia (Figure 1E,F). This hexagonal pattern minimizes boundary length and surface tension energy, thus achieving a balance between structural constraints and spatial efficiency (Yusof et al. 2022; Weaire and Rivier 1984; Ganer and Glazier 1992; Mombach and Glazier 1996). In wild‐type *Drosophila* eyes, the hexagonal pattern is established through a combination of genetic regulation and mechanical forces during development (Warren and Kumar 2023; Gallagher et al. 2022; Johnson 2021; Carthew 2007).

While the hexagonal pattern is widely regarded as a conserved structural arrangement in biological tissues, some systems have evolved alternative patterns to meet specific functional or developmental demands. For example, variations in ommatidial tiling have been observed in certain species, where non‐hexagonal configurations emerge in response to unique evolutionary forces, such as optical or structural adaptations to specific environments (Briscoe and Chittka 2001; Fincham 1984; Nilsson and Kelber 2007; Warrant and McIntyre 1993). In the *Drosophila* compound eye, wild‐type ommatidia exhibit a highly regular hexagonal arrangement (Figure 1A,B). However, certain small‐eye mutants display deviations from this pattern, with ommatidia adopting alternative configurations such as tetragonal forms (Figure 1C,D) (Kumar and Moses 2001; Choi and Benzer 1994; Wang et al. 2014; Singh et al. 2011). These observations challenge the simplistic assumption that hexagonal tiling is purely a mechanical optimization and underscore the critical role of developmental factors in shaping ommatidial arrangements.

In certain *Drosophila* mutants characterized by reduced eye size, the regular hexagonal tiling of ommatidia undergoes a marked transformation into a tetragonal arrangement (Figure 1C,D). To elucidate the underlying mechanism, we previously conducted a series of experimental investigations combining morphological observations, live imaging, and laser ablation (Hayashi et al. 2022; Togashi et al. 2024). These analyses revealed that, in small‐eye mutants, increased tissue tension along the dorsoventral (DV) axis induces pronounced vertical stretching of the compound eye epithelium during development. This mechanical deformation results in the elongation of the hexagonal ommatidial lattice along the vertical axis.

To examine whether this deformation alone is sufficient to account for the observed pattern transition, numerical simulations based on the vertex model—a mathematical framework for describing cell shape and packing—were performed under conditions of enhanced DV tension (Hayashi et al. 2022). Notably, vertical stretching of the eye field by itself failed to reproduce the hexagonal‐to‐tetragonal transition in silico, indicating that DV elongation alone is insufficient. These results suggest that the conventional vertex model lacks essential components required to capture tetragonal tiling and point to the involvement of additional, previously uncharacterized mechanical factors.

To further investigate this pattern transition, we applied a geometrical modeling approach based on the Voronoi diagram (Hayashi et al. 2022). By placing seed points at ommatidial centers and allowing each region to grow isotropically until meeting its neighbors, this model successfully recapitulated both hexagonal and tetragonal tiling patterns. This result provided a biologically plausible interpretation of Voronoi tessellation, suggesting that ommatidial patterning could be driven by an effective cellular expansion force underlying the concentric growth of ommatidia. However, this framework remains purely geometric and does not capture the temporal dynamics or force‐driven processes underlying the patterning transition.

**FIGURE 2:**
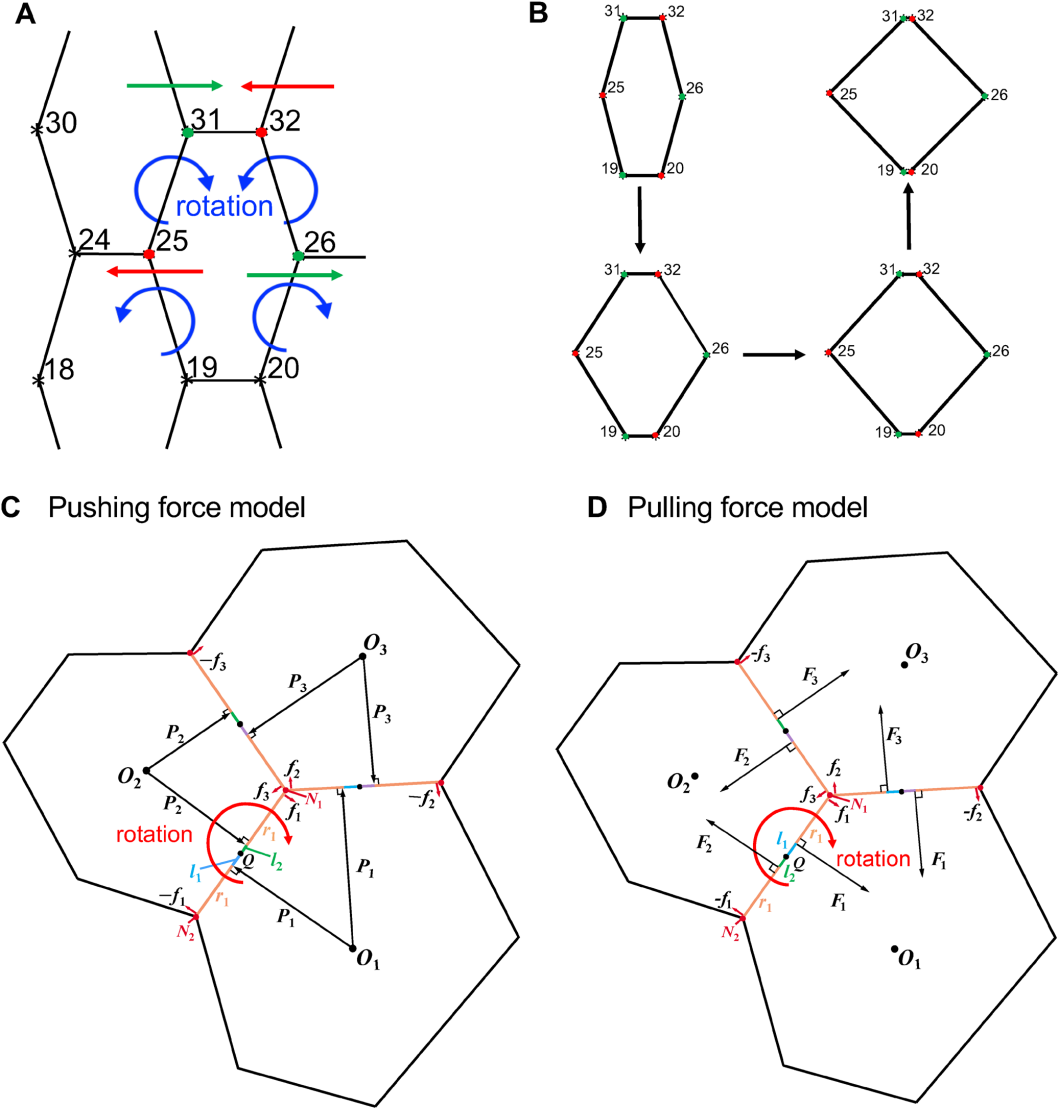
Anisotropic forces acting on the vertices. Conceptual schematics illustrating directions and mapping of forces. Note that the arrows do not indicate physical application points. (A) Schematic illustration of torque generation on oblique lateral edges. Red arrows indicate leftward forces acting on the red vertices, while green arrows indicate rightward forces acting on the green vertices. These opposing anisotropic forces generate a net torque on the oblique lateral edges connecting vertices 31–25 and 32–26, producing edge rotation as illustrated by the blue curved arrows. In contrast, the upper and lower horizontal edges connecting vertices 31–32 and 19–20 do not exhibit appreciable rotation because the opposing contributions are collinear and the effective moment arms vanish by symmetry. (B) Minimal geometric example showing how simultaneous rotation of the four oblique lateral edges converts a vertically elongated hexagon into a tetragon‐like configuration. Arrows indicate the sequence of edge rotations; vertex indices (31, 32, 25, 26, 19, 20) correspond to those in panel (A). (C) Anisotropic pushing force: This schematic represents three adjacent ommatidia, with *O*_1_, *O*_2_, and *O*_3_ denoting their centroids. Each cell edge receives radial forces from two adjacent cells, which are distributed to the vertices as shown. These forces, denoted as ***f***_1_, ***f***_2_, and ***f***_3_, form force couples (***f***_1_, −***f***_1_), (***f***_2_, −***f***_2_), and (***f***_3_, −***f***_3_), generating rotational torques on the edges. In the edge *N*_1_ *N*_2_ between *O*_1_ and *O*_2_, the distance from the midpoint *Q* to vertices *N*_1_ and *N*_2_ is *r*_1_. The distances *l*_1_ and *l*_2_ represent the moment arms for the anisotropic pushing forces ***P***_1_, ***P***_2_, which act perpendicularly to the edge *N*_1_ *N*_2_. (D) Anisotropic pulling force: The distances *l*_1_ and *l*_2_ represent the moment arms for the anisotropic pulling forces ***F***_1_, ***F***_2_, which act perpendicularly to the edge *N*_1_ *N*_2_. Note that *l*_1_ and *l*_2_ are reversed from the pushing force model.

To address this limitation, the present study focused on the nature of the mechanical forces that drive concentric ommatidial growth and mediate the observed reorganization of cell shape. Actin fibers extending radially within the primary pigment cells that constitute the ommatidia are candidates for intracellular structures that generate forces promoting the concentric growth (Johnson et al. 2008). Although the physiological function of this structure remains unclear, we revealed that this arrangement changes over time concurrently with cellular shape changes within the ommatidium (Figure 1). Thus, radial actin fibers extending within the primary pigment cells may exert some mechanical influence on the cell membrane. Although uniform pressure can induce isotropic expansion of ommatidial cells, it cannot generate rotational motion of ommatidial boundaries and is therefore insufficient to explain the transition from vertically elongated hexagons to tetragons (Figure 2A,B). Instead, a physical mechanism capable of inducing edge rotation is required.

Rotational rearrangements of ommatidial edges arise when anisotropic forces act in different directions across a shared edge, generating net torque at cell boundaries without producing net translation. While anisotropy in vertex models is often represented as orientation‐biased junctional tension or active stress, these formulations do not explicitly account for cytoplasmic force generators capable of producing boundary‐scale rotational torques. In this study, we incorporate anisotropic forces derived from radial actin organization into a vertex framework, enabling torque generation at ommatidial edges. This model successfully reproduces the experimentally observed transition from hexagonal to tetragonal tiling and provides a framework to examine how anisotropic cellular forces drive geometric reorganization of epithelial patterns.

## 2 Materials and Methods

### 2.1 Fly Strains

Standard *Drosophila* medium was used to maintain fly strains at 25°C. Male flies were used for all experiments. The following fly strains were used: *upd* ^*os1*^ (BDSC#996), *GMR‐Gal4* (BDSC#1104), *UAS‐LifeactGFP* (*UAS‐LaGFP*; BDSC#57326), and *UAS‐cpa RNAi* (BDSC#41685; VDRC#100773).

### 2.2 Live Imaging

For live imaging, a pupa was stuck on a glass slide with UV‐curable adhesive glue, and the eye was exposed by removing the pupal case of the head part. The pupa was then surrounded with silicone grease, a small amount of water was put on the eye, and the pupa was mounted with a cover slip. The specimen was analyzed with a LSM880 (Zeiss) confocal system equipped with Airyscan.

### 2.3 Vertex Model

The vertex model was originally proposed by Honda as a two‐dimensional boundary‐shortening approach to minimize the total boundary length in a given vertex network (Nagai and Honda 2001; Gibson and Gibson 2009; Fletcher et al. 2014). This model structure relies on three main elements: a polygonal network of cell patterns; a potential energy function to define tissue characteristics; and transition rules for configurations (Alt et al. 2017; Staple et al. 2010; Bi et al. 2015). The geometry of the polygonal network is defined by a set of vertex positions, denoted as ***r***_*i*_. The geometrical features of the tissue can be represented by vertices as multicellular junctions and edges as cell boundaries. The mechanical properties of the network are described by a potential function (as described in Hayashi et al. 2022)

(1) $$U\left(r_{i}\right)=\sigma \sum_{j=1}^{m}S_{j}+\kappa \sum_{j=1}^{m}{\left(A_{j}-A_{0}\right)}^{2}$$

The first term represents the interfacial energy, with the sum of the perimeters taken over all cells. $S_{j}$ is the perimeter of cell j. The second term represents the total elastic energy of area. $A_{j}$ is the area of the cell j, while $A_{0}$ is the preferred area. σ and κ are positive coefficients that influence the efficiency of perimeter shortening and area conservation, respectively. m is the cell number. For simplicity, we modeled multiple cells in a single ommatidium as one polygon in this study.

The equations of motion for the vertex positions are given by

(2) $$\eta \frac{dr_{i}}{\mathrm{dt}}=-\frac{\partial U\left(r_{i}\right)}{\partial r_{i}}$$

The friction coefficient η represents the magnitude of the friction between cells (η = 1). On the right‐hand side of Equation (2), the negative gradient of the potential energy U represents the direction of the steepest descent of energy. The vertices move according to the equation, gradually reducing the potential energy. This dynamic process drives the vertex configuration toward a stable state where the energy is minimized, ultimately resulting in a stable shape described by the vertices.

### 2.4 Vertex Model With Anisotropic Pushing Force

Before introducing the biological origin of the forces, we first describe the geometric requirement for tetragonization. A vertically elongated hexagon cannot become a tetragon‐like configuration by vertical stretching alone; instead, the four oblique lateral boundaries must rotate in a coordinated manner to reduce the side angles toward 90° (Figure 2A,B). To capture this minimal geometric requirement, we introduced edge‐associated anisotropic pushing forces that generate an effective torque on each oblique edge (Figure 2C). Importantly, at this stage $P_{1}$ and $P_{2}$ are introduced as forces that represent a directionally biased active contribution driving edge rotation. Experimental observations during developmental stages 34–40 h after puparium formation (h APF) revealed that radial actin fiber distribution was nonuniform, varying in density along cell boundaries (Figure 1B). Calculating the rotational force generated by these pushing forces from imaging data is difficult.

In the present work, anisotropic pushing force refers operationally to direction‐dependent active force generation at cell boundaries, implemented at the vertex level as edge‐associated active contributions that bias remodeling in a preferred direction. The alignment of radial actin fibers implies such a directional bias along each ommatidial boundary (Figure 1). Therefore, instead of resolving forces generated by many individual fibers, we coarse‐grained their collective effect by taking the strengthened direction as the effective direction of action on that boundary and representing it by a single resultant edge force $P_{e}$ (e.g., $P_{1}$, $P_{2}$, and $P_{3}$ in Figure 2C). In the pushing formulation, $P_{e}$ was modeled as originating from the ommatidial centroid and acting perpendicular to the edge, which provides a minimal representation of the directionally strengthened active contribution responsible for boundary remodeling. This centroid‐referenced definition is a geometric coarse‐graining, not a claim that the underlying active processes are generated at a single physical point in vivo. We used the centroid as a minimal, symmetry‐preserving reference that uniquely specifies the line of action of an effective edge‐normal contribution while avoiding additional unconstrained parameters.

The enhanced vertex model with anisotropic force is expressed as
(3)

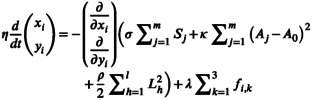




**FIGURE 3:**
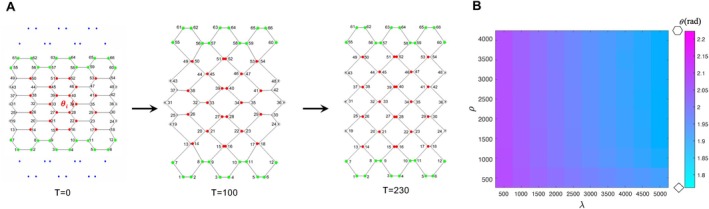
Anisotropic forces induce tetragonization in simulations. (A) Simulation of the hexagonal‐to‐tetragonal transition driven by anisotropic pushing forces at three time points. The left panel shows the initial hexagonal tiling configuration (*T* = 0). The middle panel shows the intermediate state (*T* = 100). The right panel shows the final state after deformation (*T* = 230). Green vertices are connected to blue external points via hypothetical springs that apply vertical stretching, resulting in a 1.6‐fold elongation of the field along the DV axis. Numbers indicate vertex indices used to track individual cell deformations. (B) Phase diagram showing how the vertical stretching coefficient *ρ* and the anisotropic‐force coefficient *λ* jointly determine pattern geometry. Each (*λ*, *ρ*) pair corresponds to an independent simulation. Here, we quantify hexagonality operationally by the mean side angle *θ* (radians) measured at the red vertices in the final configuration (A). The color bar indicates *θ*: smaller values (toward *π*/2≈1.571) indicate a more tetragon‐like geometry, whereas larger values (toward 2*π*/3≈2.094) indicate a more hexagon‐like geometry. Because the pushing and pulling implementations are operationally equivalent at the edge‐level force‐couple representation, the *λ–ρ* phase diagram is identical for both models.

The third term of Equation (3) introduces a vertical stretching mechanism adapted from our previous study (Hayashi et al. 2022), where *ρ* represents the spring constant that regulates the strength of the vertical stretching, $L_{h}$ denotes the length of the *h‐*th spring, and l is the total number of springs. In this framework, the uppermost and lowermost vertices are connected to external points via hypothetical springs (Figure 3A). As these springs contract, they induce an elongation of the entire field along the vertical axis. This model was adopted based on experimental observations showing that tension is specifically increased along the DV axis in the tetragonal mutant eyes (Hayashi et al. 2022). Our previous laser ablation experiments revealed that, after ablation, cells migrated significantly faster in the vertical direction in tetragonal mutant eyes than in control eyes. This indicated stronger vertical tension in mutant eyes. Therefore, we introduced spring‐based vertical constraints to represent the mechanical environment observed in vivo. In contrast, no comparable tension enhancement was reported along the horizontal axis, and thus no additional stretching was imposed on the lateral boundaries.

The fourth term introduces the anisotropic forces acting at each vertex i. Here, λ is a scaling coefficient that determines the magnitude of these anisotropic forces, while $f_{i,k}$ represents the force contribution from the *k‐*th edge connected to the *i‐*th vertex. Each vertex typically experiences rotational forces from three adjacent edges if the shape of ommatidia is not regular hexagon (Figure 2A).

The anisotropic pushing forces $P_{1}$, $P_{2}$, and $P_{3}$ (Figure 2C) represent radial forces that are initially applied to the shared edges between adjacent cells and are distributed to the vertices. For the edge between vertices $N_{1}$ and $N_{2}$, $P_{1}$ and $P_{2}$ act perpendicularly on opposite sides of the midpoint Q in opposite directions, generating a net torque of magnitude $l_{1}|P_{1}|+l_{2}|P_{2}|$, where $l_{1}$ and $l_{2}$ are moment arms and $|P_{1}|$ and $|P_{2}|$ are the force magnitudes. This torque is distributed to vertices $N_{1}$ and $N_{2}$ by a pair of equivalent forces such that the net torque is preserved. The distance between Q and $N_{1}$ and between Q and $N_{2}$ is $r_{1}$. In the simulation, $|P_{1}|$ and $|P_{2}|$ are normalized to 1, simplifying the force representation while focusing on rotational dynamics. The same procedure is applied to all three edges that are subjected to rotational forces. The anisotropic force $f_{1}$ at vertex $N_{1}$, as shown in Figure 2C, is given by

(4) $$f_{1}=\frac{\left(l_{1}|P_{1}|+l_{2}|P_{2}|\right)e_{2}}{2r_{1}}$$

The corresponding force $-f_{1}$ acts along the direction $e_{1}$, and its expression is

(5) $$-f_{1}=\frac{\left(l_{1}|P_{1}|+l_{2}|P_{2}|\right)e_{1}}{2r_{1}}$$

Here, $e_{1}$ and $e_{2}$ are unit vectors of $P_{1}$ and $P_{2}$, respectively. By modeling the net effect of distributed radial anisotropic pushing forces as a single force acting on each edge, this approach simplifies the calculation while still capturing the rotational dynamics necessary for the transition from hexagonal to tetragonal patterns.

### 2.5 Vertex Model With Anisotropic Pulling Force

In the puling force model, the anisotropic pulling forces $F_{1}$, $F_{2}$, and $F_{3}$, generated by the radial actin fibers, actively pull the cell edges inward (Figure 2D). Similar to the principle applied in the anisotropic pushing force model, the pulling forces $F_{1}$, $F_{2}$, and $F_{3}$ are applied perpendicular to the edges at specific points as defined below and distributed to the vertices as $f_{1},f_{2}$, and $f_{3}$ (Figure 2D). When the ommatidial shape is an irregular hexagon, each vertex experiences pulling forces from three adjacent edges.

The pulling force $F_{1}$ is applied perpendicular to the edge between vertices $N_{1}$ and $N_{2}$ with the moment arms $l_{1}$ and $l_{2}$ (Figure 2D). Here, $l_{1}$ and $l_{2}$ are interchanged from the pushing force model so that the pulling force produces the same rotational effect as the pushing force (Figure 2C). Along with the edge between vertices $N_{1}$ and $N_{2}$, $F_{1}$ and $F_{2}$ act perpendicular to the edge on opposite sides of the midpoint Q and in opposite directions. The distance between Q and $N_{1}$ and between Q and $N_{2}$ are defined as $r_{1}$. The same procedure is applied to all three edges that are subjected to rotational forces.

The anisotropic force $f_{1}$ acting at the vertex $N_{1}$ along the direction of $e_{1}$ is calculated as follows (Figure 2D):

(6) $$f_{1}=\frac{\left(l_{1}|F_{1}|+++l_{2}|F_{2}|\right)e_{1}}{2r_{1}}$$

where $|F_{1}|$ and $|F_{2}|$ are the magnitudes of the pulling forces in individual cells normalized to 1 (Figure 2D). The corresponding force $-f_{1}$ acting at the vertex $N_{1}$ along the direction $e_{2}$ is calculated as follows:

(7) $$-f_{1}=\frac{\left(l_{1}|F_{1}|+++l_{2}|F_{2}|\right)e_{2}}{2r_{1}}$$

Here, $e_{1}$ and $e_{2}$ are unit vectors of $F_{1}$ and $F_{2}$, respectively.

### 2.6 Coding Algorithms

All vertex model simulations in this study were implemented using custom MATLAB code. Vertex positions were updated by explicit Euler integration of the energy gradient descent described in Equation (2). The friction coefficient η was set to 1, and the time step was set to dt = 3 × 10^−5^. The initial configuration was a regular hexagonal tiling in which each cell had a side length of 10. The target cell area was set to $A_{0}$ = 360. The area and perimeter energy coefficients were set to κ = 20 and σ = 1000, respectively (Figure 3).

In the model, anisotropic forces were applied perpendicular to each edge. These forces were decomposed into equivalent force couples acting on the adjacent vertices, thereby generating rotational torques. The parameters λ and ρ (Equation 3) were systematically varied in discrete steps over the ranges 500–5000 and 500–4000, respectively.

Hexagonal index was calculated by averaging the interior angles of the selected ommatidial vertices specified in Figure 3A at the final state. Since ommatidia located at the edges of the region often exhibit irregular shapes, only centrally positioned ommatidia were considered. Additionally, since the average of all interior angles is always 120°, only the interior angles on the left and right of the ommatidia were considered.

(8) $$\theta \equiv \frac{1}{N}\sum_{i=1}^{N}\theta_{i}$$

Here, $\theta_{i}$ denotes the angle at the vertex marked with a red dot where number is indicated (Figure 3A). A regular hexagon has θ = 120° (≈2.094 rad), while a regular tetragon has θ = 90° (≈1.571 rad). Thus, larger θ indicates stronger hexagonalization, while smaller θ indicates stronger tetragonization. In Figure 3B, θ is shown in radians to visualize how λ and ρ together influence the ommatidial shape.

**FIGURE 4:**
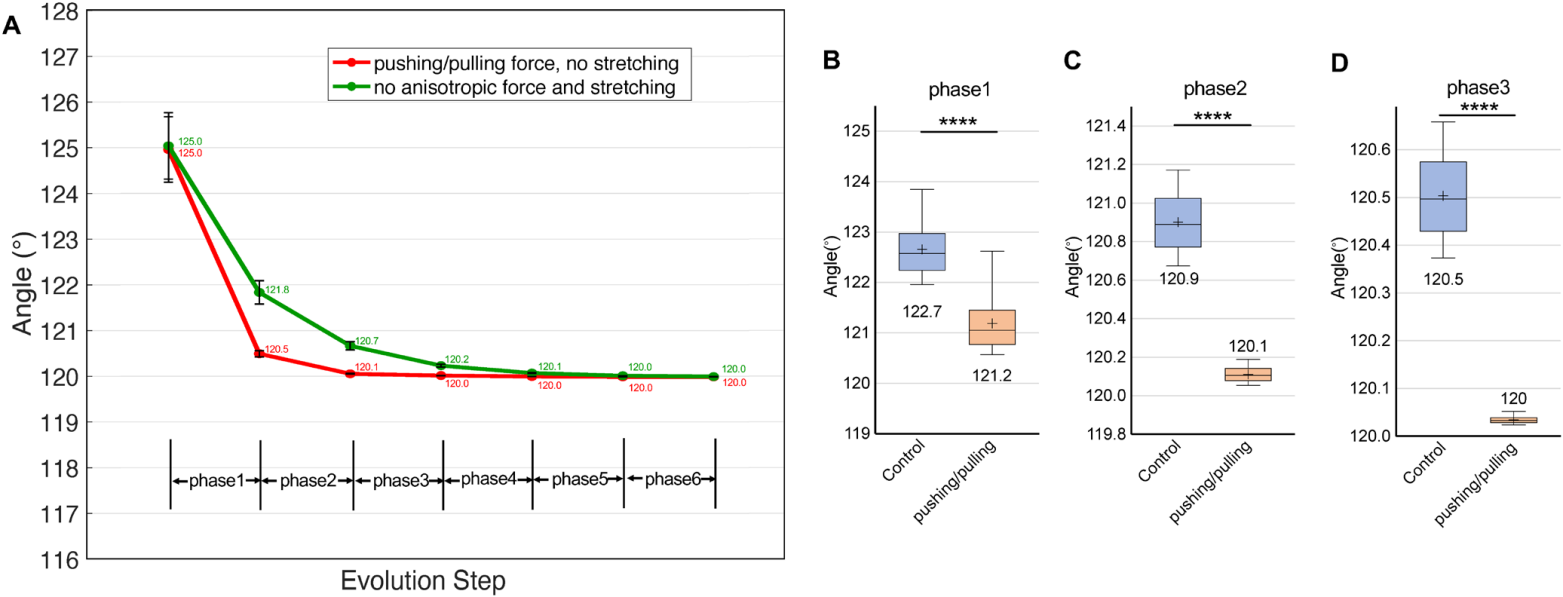
Anisotropic forces contribute to the establishment of hexagonal patterns. (A) Mean interior angles of selected red vertices (Figure 3A) over time under two conditions: anisotropic pushing/pulling forces without vertical stretching (red), control without anisotropic forces and without vertical stretching (green). Each curve represents the mean ± standard error from five independent simulations. Error bars indicate standard error at each time point. The simulation duration is divided into six consecutive phases, separated by vertical black lines. (B–D) Statistical comparison of interior angles among the three conditions in the first three phases (*n* = 50 time points). Two‐sided *t*‐test (****, ***, **, and * indicate *p <* 0.0001, 0.001, 0.01, and 0.05, respectively; NS, not significant). Mean values are indicated. Plus sign, mean; center line, median; box limits, upper and lower quartiles; whiskers, minimum to maximum.

Small geometric perturbations were applied to a regular hexagonal pattern by adding zero‐mean random variations to its side lengths, $a_{i}=a\left(1+\epsilon_{i}\right)$ (*i* = 1, …, 6), where a is the original side length and $\frac{1}{6}\sum_{i=1}^{6}\epsilon_{i}=0$ (Figure 4). The zero‐mean condition ensures that some edges become slightly longer while others become slightly shorter, without imposing a systematic overall expansion or contraction of the hexagon. We further bounded the perturbation magnitude by $|\epsilon_{i}|\leq \epsilon_{\max}\;\left(\epsilon_{\max}=0.05\right)$, so that each side length deviated from a by at most a fraction $\epsilon_{\max}$ (i.e., $a\left(1-\epsilon_{\max}\right)\leq a_{i}\leq a\left(1+\epsilon_{\max}\right)$). Using these perturbed hexagons as initial conditions, we then applied the same anisotropic forces in the absence of vertical stretching (Figure 4).

### 2.7 Data Quantification

To quantitatively evaluate the orientation of actin fibers and their geometric relationship with ommatidial boundaries, we developed a custom MATLAB‐based image‐processing pipeline. High‐resolution live‐imaging data were acquired from developing compound eyes at $t\in \left\{36,37,38,39,40,41,42\right\}$ h APF. Actin fibers were manually annotated using ImageJ to distinguish them from background and unrelated structures. The annotated fiber regions were exported as binary masks. Ommatidial boundaries were extracted in MATLAB via binary segmentation of boundary pixels and standard morphological cleanup, followed by closed‐contour tracing to obtain an ordered sequence of boundary points that follows the clockwise direction in our convention.

**FIGURE 5:**
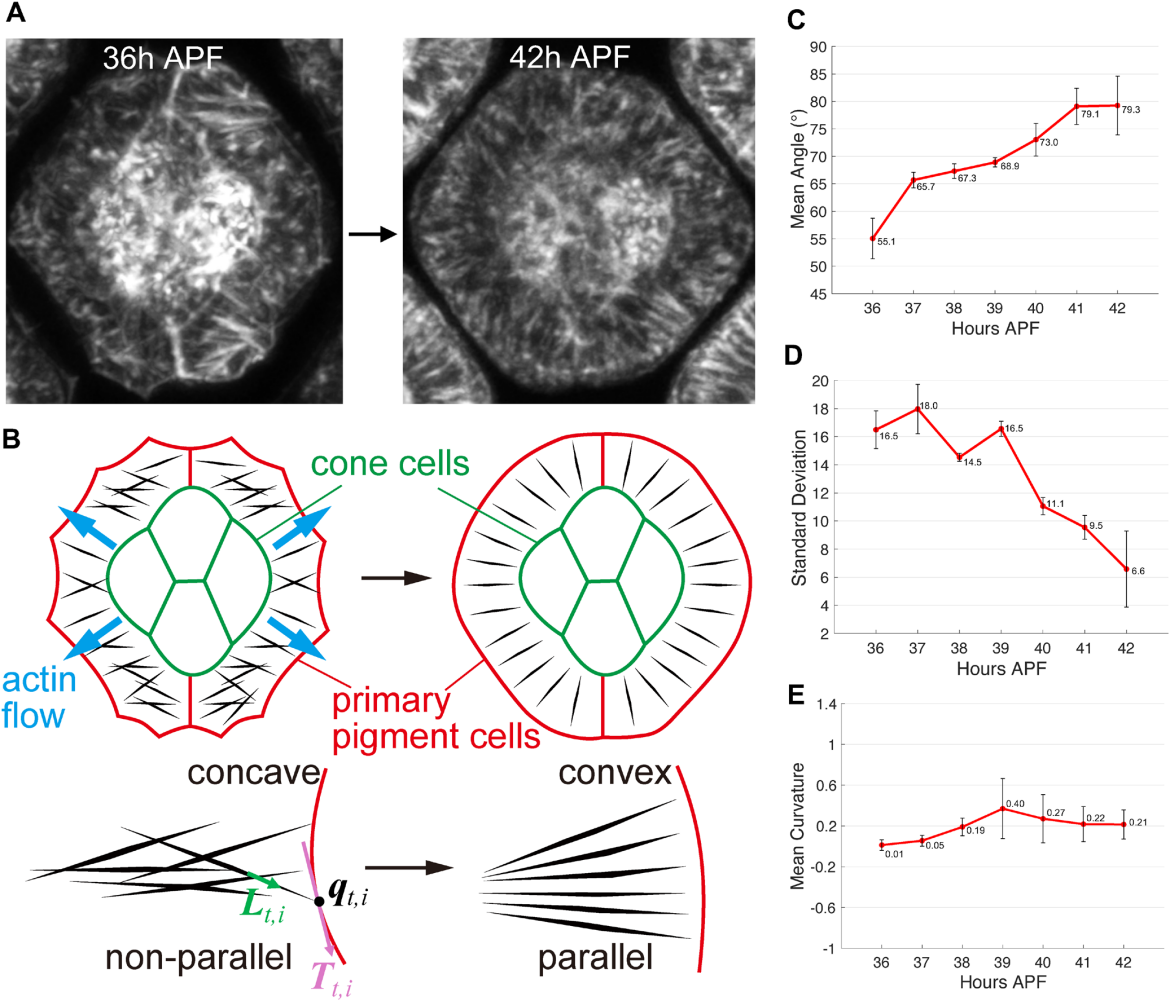
Radial actin fiber alignment and membrane curvature transition during ommatidial development. (A) High‐speed live imaging of ommatidial development at 36 h APF (Early, left) and 42 h APF (late, right). (B) Schematic representation showing actin fiber flow (blue arrows) and the transition of the ommatidial boundary (red) curvature from concave to convex over time. For a representative traced fiber segment, the outward‐pointing unit fiber direction vector is denoted by $L_{t,i}$. The intersection point between this fiber and the boundary is $q_{t,i}$, and the local unit tangent vector of the boundary at $q_{t,i}$ is denoted by $T_{t,i}$. (C–E) Quantification of the mean acute angle between $L_{t,i}$ and $T_{t,i}$ (C), the standard deviation of these acute angles across fibers at the same time point (D), and the mean curvature of the ommatidial boundary (E) over time. The horizontal axis represents time (hours APF). For (C–D), measurements were obtained from *n* = 3 samples (196 fibers in total). For (E), boundary curvature was quantified from the same *n* = 3 samples. Error bars represent the standard error of the mean (SEM).

At each time point t, we obtained $M_{t}$ traced actin fibers. For fiber i, we defined the outward‐pointing unit fiber vector $L_{t,i}$ from the cell interior toward the ommatidial boundary, as illustrated in Figure 5B. Let $q_{t,i}$ denote the intersection point between the i‐th fiber and the extracted boundary (Figure 5B). Boundary points were ordered clockwise, and we defined the local unit tangent vector $T_{t,i}$ at $q_{t,i}$ to follow the clockwise direction, computed by a centered finite‐difference approximation using the two neighboring boundary points immediately before and after $q_{t,i}$ along the ordered contour.

For each fiber, we quantified the local fiber–boundary alignment by the acute angle between the unit vectors $L_{t,i}$ and $T_{t,i}$. Since both vectors were normalized, we first computed the absolute dot product

(9) $$\;d_{t,i}=|L_{t,i}\cdot T_{t,i}|\in \left[0,1\right],$$

and then defined the acute angle $\gamma_{t,i}\in \left[0{}^{\circ},90{}^{\circ}\right]$ as

(10) $$\begin{array}{cccc} & \gamma_{t,i}=\arccos\left(d_{t,i}\right)\times \frac{180}{\pi }. & & \end{array}$$

For each time point t, we summarized the alignment across all fibers by the mean acute angle

(11) $$\begin{array}{cccc} & \gamma_{t}=\frac{1}{M_{t}}\sum_{i=1}^{M_{t}}\gamma_{t,i}, & & \end{array}$$

which is plotted as the y‐axis in Figure 5C. To quantify fiber‐to‐fiber variability in alignment at the same time point, we computed the standard deviation of $\gamma_{t,i}$, which corresponds to the y‐axis in Figure 5D.

Using the same clockwise ordering of boundary points, we computed the local signed curvature $\kappa_{t}$ at each boundary point along the extracted contour by measuring how the boundary direction changed between neighboring points using a finite‐difference approximation implemented in MATLAB. Under this sign convention, $\kappa_{t}$ > 0 indicates locally convex segments, whereas $\kappa_{t}$ < 0 indicates locally concave segments. We then computed the mean signed boundary curvature ${\bar{\kappa }}_{t}$ by averaging these local curvature values over the full contour. The resulting ${\bar{\kappa }}_{t}$ is plotted on the *y*‐axis in Figure 5E.

**FIGURE 6:**
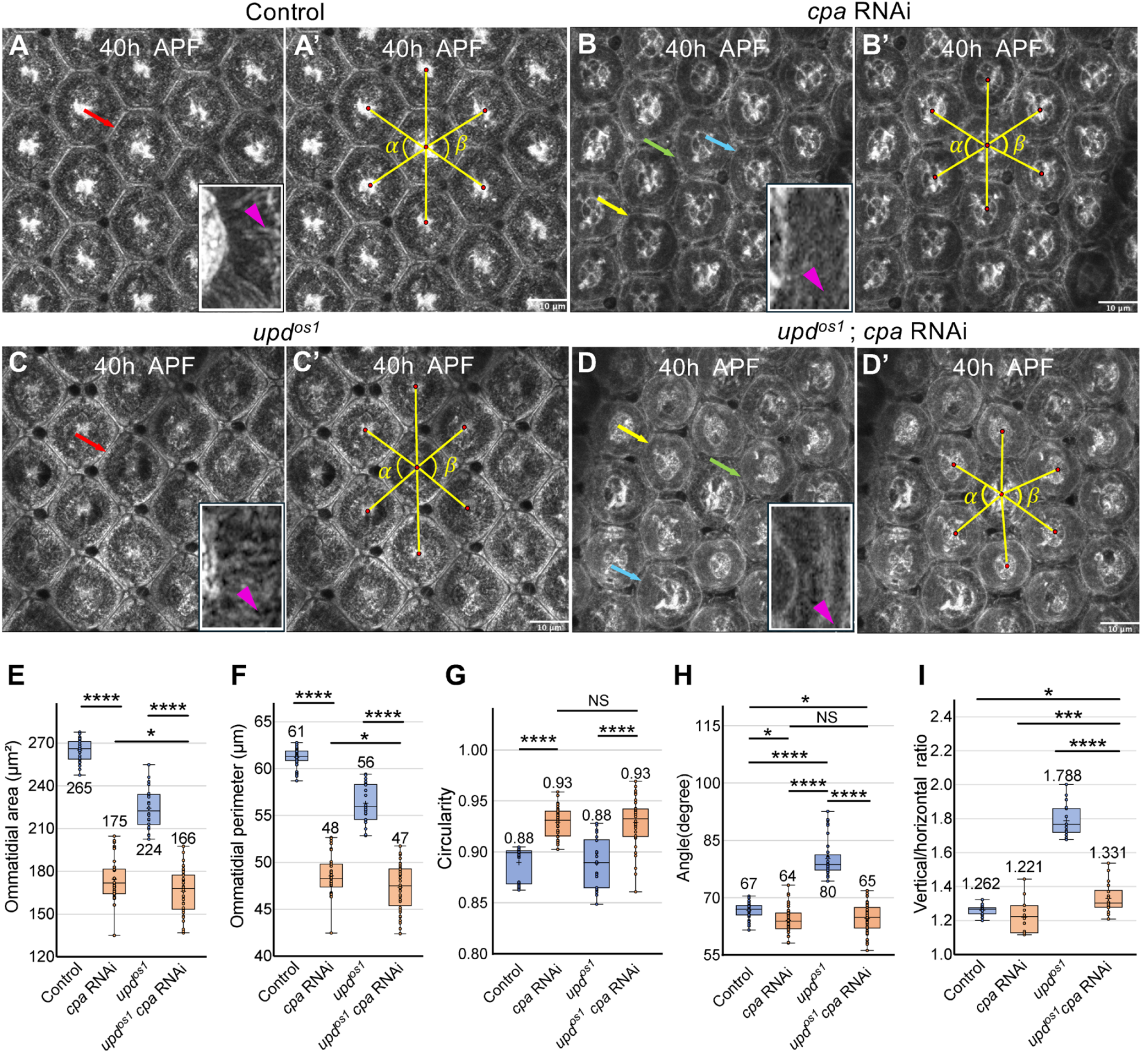
Disruption of actin fibers by *cpa* RNAi affects ommatidial patterning and primary pigment cell morphology. (A) A control eye (*GMR‐Gal4 UAS‐LaGFP*) showing a hexagonal ommatidial pattern with lattice cell contraction forming narrow inter‐ommatidial boundaries (red arrow). (B) A *cpa* RNAi eye (*GMR‐Gal4 UAS‐LaGFP* with *UAS‐cpa* RNAi) displaying an irregular pattern, with a hexagon‐like primary pigment cell (yellow arrow), a near‐circular primary pigment cell (blue arrow), and an expanded lattice cell (green arrow). (C) An *upd* ^*os1*^ mutant eye (*upd* ^*os1*^; *GMR‐Gal4 UAS‐LaGFP*) showing a tetragonal ommatidial pattern with lattice cell contraction establishing slender inter‐ommatidial boundaries (red arrow). (D) An *upd* ^*os1*^ *cpa* RNAi eye (*upd* ^*os1*^; *GMR‐Gal4 UAS‐LaGFP* with *UAS‐cpa* RNAi) exhibiting a disrupted pattern, including a hexagon‐like primary pigment cell (yellow arrow), a near‐circular primary pigment cell (blue arrow), and an expanded lattice cell (green arrow). Magnified views in the lower right corners of (A–D) highlight the presence (A, C) and absence (B, D) of radial actin fibers in typical primary pigment cells (arrowheads). (A′–D′) Measurement of vertical angles *α* and *β* formed by connecting the centers of neighboring ommatidia. Red dots indicate the centers of ommatidia. (E–I) Quantification of ommatidial area (E), perimeter (F), and circularity (G) across genotypes. Comparisons were made between control and *cpa* RNAi, between *upd* ^*os1*^ and *upd* ^*os1*^ *cpa* RNAi, and between *cpa* RNAi and *upd* ^*os1*^ *cpa* RNAi (*n* = 32, 34, 22, and 36 ommatidia for Control, *cpa* RNAi, *upd* ^*os1*^, and *upd* ^*os1*^ *cpa* RNAi, respectively). (H) Measurement of vertical angles *α* and *β* as shown in A′–D′ (*n* = 41, 37, 37, and 48 ommatidia, respectively). (I) Vertical/horizontal distance ratio between ommatidia across genotypes (*n* = 18 ommatidia). Two‐sided *t*‐test (****, ***, **, and * indicate *p <* 0.0001, 0.001, 0.01, and 0.05, respectively; NS, not significant). Mean values are indicated. Plus sign, mean; center line, median; box limits, upper and lower quartiles; whiskers, minimum to maximum.

To analyze morphological features of the ommatidia across genotypes, we quantified ommatidial area, perimeter, and circularity from confocal images (Figure 6A–D). Measurements were performed in ImageJ on manually selected ommatidia. Ommatidial area and perimeter were compared between genotypes (Figure 6E,F) using two‐sided *t*‐tests. To characterize shape regularity, circularity was computed using the following equation:

(12) $$\text{Circularity}=4\pi \times \frac{\text{Area}}{{\text{Perimeter}}^{2}}$$

This value ranges from 0 (highly irregular) to 1 (perfect circle). We calculated the ommatidial circularity and compared their distributions between genotypes to examine variations in shape regularity (Figure 6G). Furthermore, we measured the angles α and β along the vertical axis by connecting the centers of neighboring ommatidia (Figure 6A′–D′). The centroid coordinates of each ommatidium were calculated using ImageJ, and the vertical angles α and β were measured to quantitatively describe the spatial geometry of the ommatidial arrangement (Figure 6H). Using the same centroid coordinates, we also measured vertical and horizontal distances between neighboring ommatidia and calculated the ratio of vertical to horizontal distances as an index of tissue geometry (Figure 6I).

For the vertex model simulations, interior angles at the selected red vertices (Figure 3A) were calculated from the vertex coordinates at each time step as defined in Equation (8). For Figure 4A, angle values from five independent simulations were averaged at representative evolution steps to obtain the mean ± standard error under the two force conditions (with or without anisotropic pushing/pulling forces and without vertical stretching). For Figure 4B–D, angle values from 50 time points within each phase were collected for each condition and used to generate box plots and to perform two‐sided *t*‐tests. Graphs and statistical analyses for Figures 6E–I and 4 were performed using GraphPad Prism version 10.

## 3 Results

### 3.1 Considering the Role of Radial Actin Fibers

To simulate epithelial tissues, the vertex model is commonly employed, where mechanical interactions such as edge contraction are mediated by actomyosin filaments along adherens junctions (Lecuit et al. 2011; Rauzi et al. 2008; Martin et al. 2010). Unlike cell‐center models (Bi et al. 2016; Vitorino et al. 2011), which represent cells as point‐centered entities, the vertex model explicitly describes cell boundaries as edges connected by vertices. This framework enables detailed simulations of mechanical forces, including anisotropic and spatially heterogeneous force distributions, which are essential for understanding the hexagonal‐to‐tetragonal transitions observed in mutant *Drosophila* compound eyes. In this formulation, cells are characterized by their areas and perimeters, and vertex dynamics are governed by an energy functional that balances perimeter contractility and area elasticity. Although the *Drosophila* ommatidium is a 3D structure of multiple cells, membrane tension generated by actin fibers at adherence junctions primarily determines its 2D shape (Figure 1E). For simplicity, we modeled multiple cells in a single ommatidium as one polygon in this study.

Actin fibers, as key components of the cytoskeleton, regulate cellular shape by generating contractile forces at the cell cortex. We were particularly interested in the actin fibers radially arranged in the primary pigment cells during pupal development (Bhattarai et al. 2026; Johnson et al. 2008). To better understand the contribution of the radial actin fibers, we performed high‐speed live imaging at 36–42 h APF, and found that during ommatidial development, radial actin fibers initially exhibited a random orientation but progressively aligned in parallel, coinciding with a shift in membrane curvature from concave to convex (Figures 1B and 5A,B).

Radial actin fiber alignment was evaluated by representing each actin fiber as a vector pointing from the cell interior toward the boundary ($L_{t,i}$), while the corresponding tangent vector at the boundary was defined ($T_{t,i}$; Figure 5B, Section 2.7). We computed the mean angle between them in Figure 5C. The increasing trend in this metric indicates that actin fibers progressively orient toward a direction more perpendicular to the ommatidial boundary. This pattern suggests that their orientation shifts to exert forces more directly toward the ommatidial boundary, potentially generating anisotropic mechanical forces.

To further quantify the variability in radial actin fiber orientation, we computed the standard deviation of the mean angles between actin fibers and ommatidial boundaries (Figure 5D). The result exhibited a decreasing trend over time, suggesting that the radial actin fibers gradually adopt a more uniform alignment during ommatidial development.

In addition to analyzing changes in actin fiber orientation, we examined changes in membrane curvature. We extracted the ommatidial boundary from each image and computed its mean curvature (Figure 5E, Section 2.7). Positive curvature corresponds to locally convex boundary segments. Although the mean curvature remained positive and increased over developmental time, ommatidial boundaries at 36 h APF appear visually dominated by concave regions (Figure 5A). This likely reflects the influence of a small number of strongly convex segments that contribute to the averaged measure. The increase in convexity may be associated with the pulsatile behavior of the tertiary‐secondary cell border during eye development reported previously (Figure 1E; Rosa‐Birriel et al. 2024). This trend supports the hypothesis that actin fiber alignment influences cellular mechanics by generating anisotropic forces at the boundary, ultimately contributing to ommatidial rearrangement.

### 3.2 Addition of Anisotropic Force to the Vertex Model

Previous implementations of the vertex model primarily relied on vertical stretching, which successfully simulated DV elongation but failed to reproduce the full hexagonal‐to‐tetragonal transition observed in small‐eye mutants (Hayashi et al. 2022). In order for the transition from vertically elongated hexagons to tetragons to occur, the lateral ommatidial boundaries must rotate simultaneously due to some sort of rotational force (Figure 2A,B). To enable this edge rotation, we incorporated the anisotropic edge‐level active force $P_{e}$ as defined in Section 2.4 (Figure 2C).

In this formulation, the direction (clockwise vs. counterclockwise) of boundary rotation is determined by the sign of the net torque generated on each oblique lateral edge, which is set by the local geometry (Figure 2C). Because the left and right oblique lateral edges are oppositely aligned in a vertically elongated hexagon, the rotational torques become opposite, leading to coordinated counter‐rotations on the two sides that together drive tetragonization (Figure 2A). Importantly, λ acts as a scaling coefficient that modulates the magnitude of the anisotropic edge‐level forces and thus the torque amplitude, but it does not prescribe the rotation direction. This also explains why rotation is prominent for the four oblique lateral edges in Figure 2A but not for the upper and lower horizontal edges (connecting vertices 31–32 and 19–20): for oblique edges, the anisotropic contributions from adjacent ommatidia are non‐collinear and act with nonzero moment arms, producing a finite net torque, whereas for the upper and lower edges the contributions are collinear/symmetric and their effective moment arms vanish, yielding negligible net torque.

In the *Drosophila* eye, our imaging indicated that the orientation of the radial actin fibers changes from acute to perpendicular angles relative to the ommatidial boundary, and from a disordered to a regular arrangement during ommatidial morphogenesis, suggesting that the resulting active contribution to boundary remodeling is not isotropic but is preferentially strengthened along specific directions, thereby biasing remodeling dynamics (Figure 5). Because each edge is shared by two adjacent ommatidia, active contributions can arise from both sides of the same edge; accordingly, the model allows edge‐associated anisotropic forces on both sides, which are distributed to the two vertices connected by that edge, causing the rotational dynamics of the ommatidial boundaries (Figure 2C,D).

Our data do not clarify the direction or sites of actin polymerization. However, from a mechanical standpoint, there are two biologically plausible classes of anisotropic forces that may contribute to this process: pushing forces generated internally by actin polymerization and pulling forces arising from contractile actomyosin activity at the cortex. The pushing mechanism is well represented by the Brownian ratchet model (Peskin et al. 1993; Mogilner and Oster 1996; Demoulin et al. 2014), originally proposed by Peskin et al., in which thermal fluctuations intermittently displace the cell membrane, creating transient gaps that allow actin monomers to insert at the growing tip of the filament. This process leads to actin filament elongation, which exerts a forward pushing force on the membrane. In contrast, the pulling mechanism involves interactions between actin filaments and myosin II motors, which generate tension through filament sliding (Huxley and Hanson 1954; Pollard and Cooper 2009; Sellers 2000). Upon activation, myosin pulls adjacent actin filaments toward each other, producing contractile force that can drive boundary reorganization through cortical tension.

Each ommatidium is centered around four cone cells that are uniformly surrounded by two primary pigment cells (Figure 1E). Thus, the cone cell cluster is widely regarded as the geometric center of the ommatidium (Cagan and Ready 1989; Hayashi et al. 2022; Johnson and Cagan 2009). Furthermore, radial actin fibers are consistently oriented toward the cone cell cluster at 40 h APF (Figure 1B,D). These observations motivate a minimal geometric assumption that the pushing force can be approximated as originating from the ommatidial center of mass. Although this representation is a geometric abstraction, it provides a tractable framework for linking actin organization to mechanical modeling. Future investigations will be required to resolve the precise force transmission mechanisms at the level of individual actin fibers and intercellular interactions.

In both pushing and pulling formulations, we represented the net anisotropic contribution on each edge by an effective resultant force $P_{e}$ or $F_{e}$ that acts perpendicular to the edge, such that non‐collinear contributions across shared edges generate an effective torque that rotates oblique lateral boundaries and enables the hexagonal‐to‐tetragonal transition (Figure 2C,D). This centroid‐referenced, edge‐normal definition is a geometric coarse‐graining of the collective actin‐driven activity along the boundary, rather than a literal point source of force in vivo.

### 3.3 Quantification of Hexagonality Under the Anisotropic Pushing Force Model

For simplicity, we focused on the pushing model at first. To quantify how the coefficients controlling anisotropic force (λ) and vertical stretching (ρ) affect the hexagonal shape of ommatidia, we systematically analyzed their interplay by measuring the hexagonal index, or hexagonality, as represented by θ, by averaging the interior angles of the ommatidial vertices specified in Figure 3A at the final state (Section 2.6). Here, λ is a scaling coefficient that determines the magnitude of the anisotropic forces, while ρ represents the spring constant that regulates the strength of the vertical stretching (Equation 3, Section 2.4). Because the pushing and pulling implementations are operationally equivalent at the edge‐level force‐couple representation, the λ−ρ phase diagram is identical in both cases; therefore, we present a single‐phase diagram (Figure 3B). To quantify how the coefficients ρ and λ, which scale the vertical stretching and anisotropic force, respectively, affect hexagonality, we performed a systematic parameter sweep and evaluated the final ommatidial geometry.

The results suggest that both λ and ρ are required to drive the transition from vertically elongated hexagons to tetragons (Figure 3B). When λ is insufficient, the anisotropic forces are too weak to rotate the lateral boundary segments, and the final angles remained close to the hexagonal value (approaching 120°) even under strong vertical stretching. As λ increases, the active contribution strengthened, boundary rotation became progressively more pronounced, and the hexagonal index decreased toward the tetragonal value (approaching 90°).

Low values of ρ resulted in inadequate vertical stretching, which failed to sufficiently elongate the hexagons along the vertical axis. This limited elongation reduced the moment arms $l_{1}$ and $l_{2}$ for the anisotropic pushing forces $P_{1}$ and $P_{2}$, thereby weakened the anisotropic forces $f_{1}$ (Figure 2C). Without sufficient $f_{1}$, the rotational dynamics along the horizontal axis are not strong enough to form tetragons. As ρ increased, the hexagons became more elongated along the vertical axis, increasing the moment arms $l_{1}$ and $l_{2}$ to amplify $f_{1}$. This enhancement in rotational dynamics progressively reduced the hexagonal index. At higher ρ values, the hexagonal index decreased toward the tetragonal value (Figure 3B).

These findings revealed that the balance between λ and ρ is essential for achieving hexagonality in the pushing force model. ρ facilitated sufficient vertical elongation of the hexagons, increasing the moment arms $l_{1}$ and $l_{2}$ (Figure 2C) necessary for anisotropic forces to act effectively, while λ enhanced the rotational dynamics by generating stronger anisotropic forces. Together, these coefficients enabled the vertex model to replicate the experimentally observed transition from elongated hexagons to tetragons, accurately reflecting the tetragonal patterns seen in the mutant eyes.

We calibrated the simulation parameters to reflect the biomechanical properties observed in the *Drosophila* eye tissue, including appropriate values for area preservation and tension along cell borders. However, anisotropic forces (λ), which are difficult to measure, were introduced progressively to monitor their effect on cell arrangement and tiling patterns, allowing us to observe specific transformations step by step. We analyzed the model outputs in terms of tile shapes and orientations, providing us with insights into the impact of anisotropic force. By simulating vertical stretching and incorporating forces that mimic radial actin‐driven anisotropic pushing forces, the vertex model successfully demonstrated how cells transition from a hexagonal to a tetragonal pattern under mechanical stress along a specific axis.

### 3.4 Quantification of Hexagonality Under the Anisotropic Pulling Force Model

It is also possible that the anisotropic forces manifest as contractile pulling forces. This alternative hypothesis suggests that the actin fibers, rather than merely pushing the boundaries outward, could generate contractile forces pulling inward, contributing to the rearrangement of ommatidia. Actin and myosin fibers are known to generate forces through actomyosin contraction. While previous discussions focused on the possibility of radial actin fibers creating pushing forces that promote expansion and pattern transformation, it is plausible that these fibers may also generate pulling forces through their contractile activity.

Since the radial actin fibers dynamically change their position, length, and orientation throughout pupal development, calculating the net rotational force generated by their pulling force from imaging data is very difficult (Figure 5). Therefore, we adopted a simplified modeling approach in which a pulling force was introduced to produce the same mechanical effect as the pushing force model described above. Specifically, a pulling force $F_{1}$ was applied perpendicular to the edge between vertices $N_{1}$ and $N_{2}$ with moment arms $l_{2}$ and $l_{1}$ (Figure 2D). Here, $l_{1}$ and $l_{2}$ were interchanged relative to the pushing force model (Figure 2C). The same procedure was applied to all six edges. However, as shown in Figure 2A, only the four oblique lateral edges generated a nonzero net torque and thus produced rotational motion, whereas the upper and lower edges (connecting vertices 31–32 and 19–20) remained essentially non‐rotating because the torques canceled by symmetry. As in the pushing force model, simulations based on this pulling force formulation successfully reproduced the hexagonal‐to‐tetragonal transition observed under the mutant condition (Figure 3A,B).

### 3.5 Experimental Validation of Anisotropic Cellular Force Hypothesis

Regardless of whether radial actin fibers generate pushing or pulling forces, their functional contribution can be directly tested by eliminating these structures. To this end, we focused on *capping protein alpha* (*cpa*), an actin‐binding protein required for radial actin fiber formation (Funk et al. 2021; Delalle et al. 2005). Eye‐specific RNAi against *cpa* (*cpa* RNAi) effectively eliminated radial actin fibers in both control and *upd* ^*os1*^ backgrounds (Figure 6A–D). The *upd* ^*os1*^ mutant is a small‐eye mutant in which adult ommatidia exhibit a characteristic tetragonal tiling pattern, and the pupal eye epithelium displays enhanced DV tension and vertical elongation (Figure 1C,D; Hayashi et al. 2022).

In control eyes, ommatidia displayed a regular hexagonal arrangement with minimal interommatidial space (Figure 6A). In contrast, *cpa* RNAi caused the expansion of interommatidial areas formed by lattice cells and disrupted the regular hexagonal tiling (Figure 6B). This change was accompanied by a reduction in radial actin fibers and a more circular morphology of ommatidia (Figure 6E–G).

In *upd* ^*os1*^ mutant eyes, a characteristic tetragonal ommatidial pattern was observed (Figure 6C). However, in *upd* ^*os1*^ *cpa* RNAi eyes, the tetragonal tiling pattern was no longer maintained (Figure 6D). Instead, the ommatidial organization closely resembled that of *cpa* RNAi eyes in the control background, including reduced ommatidial size and increased circularity (Figure 6E–G).

Since the shape of the ommatidial boundaries became curved upon *cpa* RNAi, it was difficult to calculate the hexagonal index, which is defined based on cell morphology. To quantify ommatidial arrangement independently of boundary curvature, we instead measured the average vertical angles formed by connecting the centers of neighboring ommatidia (Figure 6A′–D′, Section 2.7). This metric reflects the spatial arrangement of ommatidia rather than their individual shape. In a hexagonal arrangement, this angle approaches 60°, whereas in a tetragonal arrangement it approaches 90°. The average vertical angle in *upd* ^*os1*^ mutant eyes was significantly larger than in control, *cpa* RNAi, and *upd* ^*os1*^ *cpa* RNAi eyes (Figure 6H). Only minor differences were observed among control, *cpa* RNAi, and *upd* ^*os1*^ *cpa* RNAi groups.

Together, these results demonstrate that depletion of radial actin fibers suppresses the tetragonal tiling phenotype of the *upd* ^*os1*^ mutant and disrupts regular ommatidial geometry, supporting the hypothesis that radial actin fibers are required for maintaining anisotropic forces underlying ommatidial patterning.

To address the possibility that *cpa* RNAi might also reduce membrane tension and thereby affect the vertical stretching enhanced by the *upd* ^*os1*^ mutation, we quantified the ratio of vertical to horizontal distances between neighboring ommatidia. This vertical/horizontal ratio was reduced in *upd* ^*os1*^ *cpa* RNAi compared with *upd* ^*os1*^ alone, yet remained higher than in control and *cpa* RNAi eyes (Figure 6I). These measurements indicate that, although tissue‐level vertical tension contributes to ommatidial elongation, radial actin fiber–mediated anisotropic forces are essential for the hexagonal‐to‐tetragonal pattern transition. Together, these results support a model in which radial actin fiber–mediated anisotropic forces cooperate with tissue‐wide tension to regulate the hexagonal‐to‐tetragonal transition and maintain ommatidial geometry.

### 3.6 The Potential Roles of Anisotropic Forces in Maintaining Hexagonal Patterns in Control Conditions

The tetragonal mutant background used in this study does not necessarily reflect physiological conditions. This raises the question of what roles anisotropic forces driven by radial actin fibers might play under wild‐type conditions. To address this, we performed additional simulations using mildly perturbed hexagonal patterns to mimic the ommatidial shapes observed in wild‐type eyes. Specifically, we introduced small geometric perturbations to initially regular hexagons by applying zero‐mean random variations to their side lengths. The same anisotropic forces were then applied in the absence of vertical stretching (Section 2.6).

The time series of interior angles (see Figure 3A) at the selected red vertices were calculated (Figure 4A). The results demonstrated that, in the presence of anisotropic pushing or pulling forces, the shapes of the ommatidia more quickly approached regular hexagons. The accelerating effect of anisotropic forces was most prominent during the early simulation phases (Figure 4B–D), after which all conditions gradually converged toward the characteristic angles of regular hexagons. This suggests that anisotropic forces stabilize hexagonal tile patterns in a wild‐type background and drive the hexagonal‐to‐tetragonal transition in a tetragonal mutant background.

In the pushing force model, anisotropic forces were applied radially and perpendicularly to the cell edges, so the moment arms depended solely on the geometric deviation from a regular hexagon. As the ommatidial shape evolved toward a regular hexagon, the moment arms approached zero, leading the vertex‐level anisotropic forces to gradually vanish. The pulling force model was designed to have the same effect as the pushing model and showed essentially the same results (Figure 4).

## 4 Discussion

### 4.1 Extending the Vertex Model to Incorporate Cytoplasmic Torque Generation

As highlighted in previous studies (Ishihara and Sugimura 2012; Briñas‐Pascual et al. 2024) and further supported by discrepancies between classical vertex model simulations and experimental observations (Hayashi et al. 2022; Togashi et al. 2024), morphogenetic transformations that involve symmetry breaking, directional rearrangements, and actively generated forces require careful specification of the relevant mechanical ingredients and their biological origins within the modeling framework.

In its conventional form, the vertex model represents epithelial mechanics through an energy function that typically includes perimeter and area terms, capturing effective junctional contractility and area elasticity (Equation 1). Importantly, vertex models are not intrinsically restricted to passive mechanics. A substantial body of work has extended the vertex framework to incorporate active stresses and anisotropy, for example, via polarized junctional tensions, time‐dependent contractility, or edge‐/angle‐dependent active terms, and these extensions have successfully accounted for directional tissue remodeling in multiple contexts (Honda et al. 2008; Sato et al. 2015; Suzuki et al. 2017).

In practice, the choice of active terms should be guided by the specific force‐generating structures and regulatory mechanisms supported by experiments in the system of interest. While many existing implementations focus on junction‐centered active tensions, they are not primarily formulated to represent cytoplasmic force generators that act through rotational torques at the scale of a cell boundary.

Here, anisotropic force refers specifically to the centroid‐referenced, edge‐normal active resultants $P_{e}$ and $F_{e}$ (Equation 3, Section 2.4), introduced to generate an effective torque for edge rotation rather than angle‐dependent line‐tension formulations. In the *Drosophila* compound eye, our experimental observations point to radial actin organization within primary pigment cells as a cytoplasmic structure capable of generating such anisotropic forces. The central contribution of the present work is to integrate this cytoplasmic mechanical component into a vertex‐based description by parameterizing its net mechanical effect as an active torque‐producing term, implemented here in both anisotropic pushing and anisotropic pulling formulations. This provides a minimal, experimentally grounded mechanism that links cytoplasmic actin architecture to vertex‐level rotational dynamics driving the hexagonal‐to‐tetragonal transition, thereby complementing prior junction‐centered active/anisotropic formulations.

Vertex models are often implemented within an energy minimization framework, in which mechanical equilibrium is determined by minimizing an effective energy functional. Many studies have extended this framework to incorporate viscous and time‐dependent mechanical responses, allowing vertex‐based models to describe dynamic morphogenetic processes. For example, viscosity was integrated into vertex dynamics (Ujihara et al. 2010; Okuda et al. 2015), while active mechanical behaviors were incorporated into vertex formulations to model tissue remodeling and collective cell movements (Honda et al. 2008; Sato et al. 2015; Suzuki et al. 2017). Furthermore, analyses of vertex models under oscillatory deformation have revealed viscoelastic behavior consistent with standard rheological models in both solid‐like and fluid‐like regimes (Tong et al. 2022). These developments highlight that vertex models can capture time‐dependent rheology, which is essential for describing directional cell rearrangements and tissue dynamics.

Additionally, the assumption that cell–cell junctions act purely as passive mechanical elements oversimplifies the role of cell adhesion dynamics, which are actively regulated by molecular processes (Lecuit and Yap 2015). Together, these considerations motivate continued development of vertex‐based and related modeling frameworks that integrate experimentally supported sources of active force generation, including cytoplasmic mechanical components, and their appropriate dynamical interpretations for epithelial patterning and tissue morphogenesis.

### 4.2 Anisotropic Forces as a Key Driver of Structural Transitions

In the wild‐type ommatidia, both the geometric configuration and the distribution of mechanical forces are highly symmetric, preserving a stable hexagonal pattern. Under this symmetry, anisotropic forces do not generate rotational dynamics but instead contribute to stabilizing the hexagonal arrangement. Consistently, simulations with small perturbations to an initially hexagonal configuration rapidly relaxed back to regular hexagons under anisotropic forces in the absence of vertical stretching (Figure 4).

In contrast, in mutant eyes, enhanced tension along the DV axis elongates ommatidia and breaks the geometric symmetry of the lattice. This deformation alters the spatial relationship between anisotropic forces acting across shared edges, causing them to become non‐collinear. As a result, an imbalance in force vectors generates net torque at cell boundaries, initiating edge rotation and driving the transition from elongated hexagons to tetragonal patterns (Figure 2).

In contrast to the pushing force model, the force in the pulling force formulation is not assumed to be directed toward the ommatidial center. Rather than representing a specific mechanism, the pulling force model serves as a geometrically equivalent formulation that produces the same effective edge rotation as the pushing model. This provides a minimal alternative description of anisotropic edge‐normal forces without constraining the directionality to a particular intracellular architecture. Ongoing experimental analyses of the complex spatial organization and dynamics of radial actin fibers will enable more precise mapping between these model assumptions and actual cytoplasmic force distributions. The pulling force formulation introduced here therefore offers a useful theoretical framework for interpreting such future quantitative measurements.

Morphogenetic processes such as cell intercalation, elongation, and tissue reshaping commonly involve anisotropic mechanical forces, which provide directional cues essential for symmetry breaking and large‐scale structural transitions. In *Drosophila* germband extension, Rauzi et al. demonstrated that planar‐polarized cortical tension, generated by anisotropic myosin II distribution, is sufficient to drive convergent extension (Rauzi et al. 2008). Similarly, during vertebrate neural tube closure, planar cell polarity signaling induces anisotropic actomyosin contractility along mediolateral junctions, leading to apical constriction and epithelial folding (Nishimura et al. 2012).

Together, these examples place the present findings within a broader framework in which anisotropic mechanical forces act as drivers of symmetry breaking and tissue remodeling across diverse morphogenetic contexts. In this study, we demonstrated how such anisotropic forces, when combined with geometry‐dependent torque generation, can be mechanistically linked to a specific structural transition at the scale of the ommatidial lattice. This provides a concrete example of how cytoplasmic force organization can be integrated into vertex‐based descriptions to explain tissue‐level pattern transformations.

### 4.3 The Multi‐Cellular Model Simulates Cellular Interactions and Morphogenesis

A limitation of the present model is that each ommatidium—comprising up to 6 ommatidial cells and at least 12 surrounding lattice cells at the apical surface—is represented as a single polygon. To fully capture cellular morphogenesis in the compound eye, a more detailed multi‐cellular model that explicitly includes all ommatidial and lattice cells will ultimately be required.

While such multi‐cellular frameworks hold strong potential for integrating experimental data, including actin fiber alignment or externally applied mechanical stretching, translating these complex biological inputs into accurate computational simulations remains challenging. Direct comparison between simulation outcomes and experimental observations is further complicated by variability in experimental conditions and current computational constraints. Moreover, the interactions among multiple force‐generating mechanisms—such as cortical tension, anisotropic actin forces, and tissue‐level stresses—are not yet fully understood.

Future studies should therefore aim to refine multi‐cellular models to better reproduce the hexagonal‐to‐tetragonal transition observed in mutant *Drosophila* eyes, particularly by explicitly incorporating anisotropic forces arising from actin fiber organization.

### 4.4 The Bubbly Vertex Model as a Potential Framework for Ommatidial Patterning

Another limitation of the present model is that it does not explicitly represent curved cell membranes, as each cell boundary is approximated by straight line segments in the conventional vertex model. The bubbly vertex model overcomes several of these limitations by explicitly incorporating curved cell boundaries and force‐driven mechanics (Ishimoto and Morishita 2014).

This advanced formulation enables a more realistic representation of cellular interfaces and significantly improves the accuracy of tissue dynamics simulations. By explicitly modeling membrane curvature, the bubbly vertex model offers deeper insights into how mechanical forces shape tissue patterning and morphogenesis. Its ability to capture surface tension effects and curvature‐dependent force generation further highlights its broad applicability to a wide range of developmental processes.

Complementary to bubbly vertex approaches, deformable‐cell models provide an alternative route for treating curved and highly deformable cell boundaries, and have recently been used to capture complex cell shapes and 3D tissue mechanics (Runser et al. 2024).

A promising direction for future research is to integrate the curvature‐based mechanics into a multi‐cellular framework. Combining these approaches could provide a more comprehensive platform for simulating complex tissue dynamics, particularly in systems involving heterogeneous cell populations and multiple force‐generating mechanisms. Incorporating diverse cell types and anisotropic forces into a multi‐cellular model would be further strengthened by adopting curvature‐dependent mechanics.

Integrating such a hybrid model with experimental data has the potential to yield deeper insights into how membrane curvature and mechanical forces interact to drive tissue morphogenesis. By extending the scope beyond a single pattern transformation, this framework could contribute to a broader understanding of how distinct force‐driven mechanisms cooperate to shape multicellular structures during development.

## Author Contributions

T.Z., S.R.D. and M.S. conceived and designed the experiments. T.Z. and M.S. formulated the mathematical models. T.Z. performed theoretical analysis, coding, and numerical experiments. S.R.D., C.L., and W.R. performed biological experiments. T.Z., S.R.D., and M.S. acquired, analyzed, and interpreted the data. T.Z. and M.S. wrote the manuscript.

## Funding

This work was supported by the Grant‐in‐Aid for Scientific Research (A) and (B) and the Grant‐in‐Aid for Transformative Research Areas (A) from MEXT (22H05621, 22H05169, 22F22073, 22F32073, 23K21312, 24H00188, 24H01396, 25K02282, and 25K22467 to M.S.).

## Conflicts of Interest

The authors declare no conflicts of interest.

## Data Availability

The custom code newly generated in this study is available online (https://github.com/satouma7/AnisotropicForce).
